# Assessment of Fatigue and Quality of Life in Multiple Sclerosis Patients: A Cross-Sectional Study

**DOI:** 10.1192/j.eurpsy.2023.670

**Published:** 2023-07-19

**Authors:** S. Chemingui, M. Mersni, I. Youssef, I. Yousfi, S. Ernez, N. Mechergui, D. Brahim, G. Bahri, H. Ben Said, N. Ladhari

**Affiliations:** Department of Occupational Medicine, Charles Nicolle Hospital of Tunis, Tunis, Tunisia

## Abstract

**Introduction:**

Multiple sclerosis (MS) is a chronic inflammatory disorder of the central nervous system that is associated with a range of devastating symptoms including fatigue. In addition, the accumulation of disability that occurs in most MS patients can have a detrimental effect on their quality of life.

**Objectives:**

To assess fatigue and quality of life in patients with MS.

**Methods:**

Descriptive cross-sectional study that interested MS patients referred to the occupational pathology consultation of Charles Nicolle Hospital, during the period from 1 July 2020 to 30 September 2022. The data collected concerned socio-demographic and occupational characteristics. The impact of MS on quality of life was studied using the SF-12 quality of life scale. Fatigue was assessed by the Fatigue Severity Scale (FSS).

**Results:**

Twenty-six cases of MS were identified. The mean age was 38 ± 9 years with a sex ratio (M/F) of 0.3. The average occupational seniority was 11 ± 8 years. The health sector was the most represented (23%, n= 6) followed by the transport sector (19%, n= 5). The main occupations were manual workers (31%), drivers, and administrative agents (19% each). The occupational constraints were physical in 44% of cases and psychological in 24% of cases. Fourteen patients (87% of the cases) lost their jobs because of the disease. The decision on occupational fitness was definitive incapacity in 44% of cases. The mean FSS score was 4±1.74. Sixteen patients (62%) had moderate fatigue (FSS 36-52), eight patients (31%) had mild fatigue (FSS <36) and two patients (8%) had severe fatigue (FSS >52). In addition, the mean scores for the physical and mental components of perceived health were estimated to be 37.04±7.67 and 44.93±7.23, respectively. The mean global score (SG) of SF12 was 40.98±7.23. The majority of patients (92%, n=24) had an average quality of life (SG- SF12 between 30 and 60) and two patients (8%) had a poor quality of life (SG <30).

**Conclusions:**

The impact of MS on the socio-professional quality of life of patients was noted in the majority of cases. It is therefore imperative to improve the care of our patients on both the physical and psychological levels.

**Disclosure of Interest:**

None Declared

